# Transcriptome analysis of *Shank3*-overexpressing mice reveals unique molecular changes in the hypothalamus

**DOI:** 10.1186/s13041-018-0413-4

**Published:** 2018-11-27

**Authors:** Chunmei Jin, Hyojin Kang, Shinhyun Kim, Yinhua Zhang, Yeunkum Lee, Yoonhee Kim, Kihoon Han

**Affiliations:** 1 Department of Neuroscience, College of Medicine, Korea University, 73, Inchon-ro, Seongbuk-gu, Seoul, 02841 South Korea; 2 Department of Biomedical Sciences, College of Medicine, Korea University, Seoul, 02841 South Korea; 3 Division of National Supercomputing, KISTI, Daejeon, 34141 South Korea

**Keywords:** Shank3, Hypothalamus, Transcriptome

## Abstract

Various mutations in the SH3 and multiple ankyrin repeat domains 3 (*SHANK3*) gene are associated with neurodevelopmental and neuropsychiatric disorders. Thus far, synaptic abnormalities in multiple brain regions, including the hippocampus, prefrontal cortex, striatum, and ventral tegmental area, have been investigated in several lines of *Shank3* mutant mice. However, although some reports have shown loss and gain of body weight in *Shank3* knock-out and overexpressing transgenic (TG) mice, respectively, the potential functions of Shank3 in the hypothalamus, a brain region critically involved in energy intake and expenditure, are unknown. Hence, we first characterized endogenous *Shank3* mRNA and protein expression in the hypothalamus of adult wild-type mice. Thereafter, we performed transcriptome analysis (RNA-sequencing) in the hypothalamus of adult *Shank3* TG mice which mildly overexpress Shank3 proteins. By comparing the 174 differentially expressed genes in the hypothalamus with those previously reported in the striatum and medial prefrontal cortex (mPFC) of *Shank3* TG mice, we found that 159 were hypothalamus-specific while only 15 were also observed in either the striatum or mPFC. Furthermore, gene set enrichment analysis of the RNA-sequencing analysis revealed that ribosome-related genes were enriched especially in the up-regulated genes of *Shank3* TG hypothalamus, which is in contrast to the results of the *Shank3* TG striatum and mPFC analyses, where ribosome-related genes were enriched in the down-regulated genes. Beyond revealing endogenous *Shank3* mRNA and protein expression in the hypothalamus, our results suggest unique molecular changes in the hypothalamus of *Shank3* TG mice compared with those in the striatum and mPFC.

**Electronic supplementary material:**

The online version of this article (10.1186/s13041-018-0413-4) contains supplementary material, which is available to authorized users.

## Main text

Deletions, duplications, and point mutations in the SH3 and multiple ankyrin repeat domains 3 (*SHANK3*) gene, encoding excitatory postsynaptic core scaffolding proteins, are causally associated with numerous neurodevelopmental and neuropsychiatric disorders. Several mouse lines of knock-out, knock-in, overexpression, and viral knock-down for the *Shank3* gene have been generated and characterized, which has provided important insights into the neuronal pathophysiology of *SHANK3*-associated brain disorders. Specifically, detailed biochemical and synaptic abnormalities in various brain regions of the mice, including the prefrontal cortex [1–3], hippocampus [4–6], striatum [7–10], and ventral tegmental area [11], have been investigated. The hypothalamus is a brain region critical for regulating energy intake and expenditure [12]. Notably, reduced body weight of a line of *Shank3* knock-out mice was reported [13]. Moreover, *Shank3* transgenic (*Shank3* TG) mice which mildly overexpress Shank3 proteins (by approximately 50%) showed increased body weight and food intake [5]. Therefore, it is conceivable that Shank3 may have some functions in the hypothalamus; however, this has not been investigated thus far. In the present study, we aimed to understand expression and molecular functions of Shank3 in the hypothalamus, by performing transcriptome (RNA-sequencing, RNA-seq) analysis in the hypothalamus of adult (10 to 12-week old) *Shank3* TG mice (Additional file 1).

**Fig. 1:**
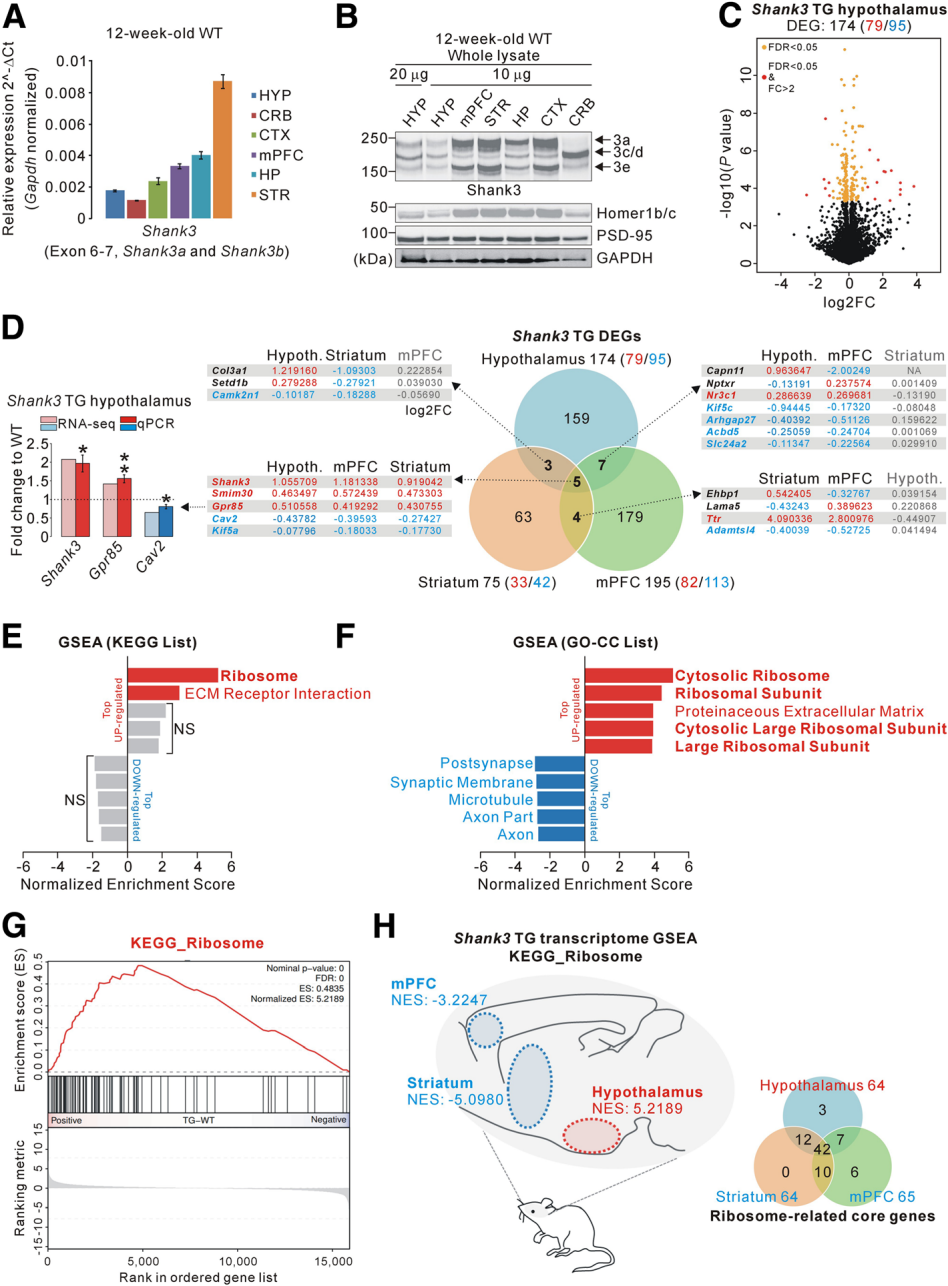
Characterization of *Shank3* expression in the wild-type hypothalamus, and transcriptome analysis of the hypothalamus in *Shank3* transgenic mice. **a** qRT-PCR results showing relative expression levels of *Shank3* mRNA in various brain regions of adult wild-type (WT) mice. HYP, hypothalamus; CRB, cerebellum; CTX, cortex; HP, hippocampus; STR, striatum. **b** Western blot images showing relative expression levels of Shank3 (3a, 3c/d, 3e isoforms), Homer1b/c, and PSD-95 proteins in various brain regions of adult WT mice. The amount of total proteins loaded in each well is indicated. **c** Volcano plot for the hypothalamic RNA-sequencing (RNA-seq) analysis of adult *Shank3* TG mice. Differentially expressed genes (DEGs), defined by FDR < 0.05, are shown as orange (FC < = 2) and red (FC > 2) circles. FC, fold change. The complete lists of the RNA-seq analysis and DEGs are provided in Additional file 2: Tables 1 and 2. **d** The Venn diagram shows the numbers of common DEGs among the hypothalamus, striatum, medial prefrontal cortex (mPFC) of *Shank3* TG mice. For the common DEGs, the log2FC values for each brain region are shown. The bar graph shows qRT-PCR results for *Shank3*, *Gpr85*, and *Cav2* in the *Shank3* TG hypothalamus. **e**, **f** The bar graphs show normalized enrichment scores (NESs) of the gene set enrichment analysis (GSEA) on the Kyoto Encyclopedia of Genes and Genomes (KEGG, E) and Gene Ontology-Cellular component (GO-CC, F) gene sets for the *Shank3* TG hypothalamus RNA-seq analysis. Significant gene sets (FDR < 0.05) are highlighted in red and blue for up-regulated and down-regulated genes, respectively. The complete lists of the GSEA are provided in Additional file 2: Table 3. **g** The enrichment plot of RNA-seq analysis of *Shank3* TG hypothalamus of the KEGG ribosome gene set. **h** The diagram shows opposite directional NES of the GSEA on KEGG ribosome gene set for the *Shank3* TG hypothalamus compared with striatum and mPFC. Blue represents negative NES value (i.e., down-regulated genes), while red represents positive NES value (i.e., up-regulated genes). The Venn diagram shows the numbers of common ribosome-related core genes among the hypothalamus, striatum, and mPFC of *Shank3* TG mice. The complete lists of the ribosome-related genes are provided in Additional file 2: Table 4. Data are presented as mean ± SEM. *P < 0.05 and **P < 0.01 (unpaired two-tailed Student’s *t*-test)

We initially characterized the expression of endogenous *Shank3* mRNA and protein in the hypothalamus because there was a report suggesting a lack of *Shank3* expression in the hypothalamus [2]. In qRT-PCR experiments using primers targeting exon 6–7 of *Shank3* (thus detecting *Shank3a* and *Shank3b* isoforms), *Shank3* mRNA levels in the hypothalamus were relatively lower than those in the cortex, medial prefrontal cortex (mPFC), hippocampus, and striatum, but higher than those in the cerebellum of adult male wild-type (WT) mice (Fig. 1a). Consistent with the mRNA expression pattern, protein levels of Shank3 and its direct binding partner Homer1b/c in the hypothalamus were relatively lower than those in other brain regions of adult male WT mice (Fig. 1b). Based on the expression of endogenous *Shank3* in the hypothalamus, we next investigated molecular changes in the hypothalamus of *Shank3* TG mice by performing a RNA-seq analysis of hypothalamic tissue from adult male WT and *Shank3* TG mice (Additional file 2: Table 1). After applying adjusted *P* values (< 0.05, Benjamini–Hochberg correction) to the transcriptome analysis, we identified 174 differentially expressed genes (DEGs) (79 up-regulated and 95 down-regulated) in the *Shank3* TG hypothalamus compared with the WT hypothalamus (Fig. 1c and Additional file 2: Table 2). Thereafter, we attempted to understand the specificity of the *Shank3* TG hypothalamic DEGs, by comparing them to DEGs from two age-matched *Shank3* TG RNA-seq studies recently published by our group. One was from the striatum (75 DEGs; 33 up-regulated and 42 down-regulated) [14], and the other was from the medial prefrontal cortex (mPFC) (195 DEGs; 82 up-regulated and 113 down-regulated) of adult *Shank3* TG mice [15]. We observed that the majority of DEGs found in each brain region were brain region-specific (Fig. 1d). Specifically, among the 174 DEGs of the *Shank3* TG hypothalamus, 159 were hypothalamus-specific while only 15 were shared with either the striatum or mPFC. Notably, five genes were commonly altered in all three brain regions of *Shank3* TG mice compared with those of the WT mice (Fig. 1d), among which we validated the changes of *Shank3*, G protein-coupled receptor 85 (*Gpr85*), and Caveolin 2 (*Cav2*) in the *Shank3* TG hypothalamus by qRT-PCR experiments (Fig. 1d, left panel).

To understand representative biological pathways or functions of the 174 DEGs in the *Shank3* TG hypothalamus, we performed Gene Ontology (GO) and Kyoto Encyclopedia of Genes and Genomes (KEGG) pathway analyses. However, we could not find significant terms in any category of the analyses, suggesting that the 174 DEGs may be too heterogeneous to be grouped into certain biological pathways or functions. Therefore, instead of focusing on the DEGs, we next performed gene set enrichment analysis (GSEA) of the RNA-seq analysis. GSEA is more useful to identify meaningful molecular signatures based on broader or overall expression changes in the transcriptome, regardless of fold change and statistical significance of each gene [14, 15]. We applied three different groups of gene sets (Hallmark, KEGG, and GO gene sets) to the hypothalamic RNA-seq analysis. For the Hallmark gene sets, “Epithelial mesenchymal transition” was significantly represented by the up-regulated genes of the *Shank3* TG hypothalamus (Additional file 2: Table 3). For the KEGG gene sets, “Ribosome” and “ECM receptor interaction” were enriched by the up-regulated genes of the *Shank3* TG hypothalamus (Fig. 1e, g and Additional file 2: Table 3). Lastly, for the GO gene sets, “Cytosolic ribosome” and “Ribosomal subunit” were enriched by the up-regulated genes, while “Postsynapse” and “Synaptic membrane” were represented by the down-regulated genes of the *Shank3* TG hypothalamus (Fig. 1f and Additional file 2: Table 3). Collectively, these results indicate that ribosome-related genes are enriched especially in the up-regulated genes of the *Shank3* TG hypothalamus, which is reminiscent of our previous GSEA of the RNA-seq analyses of the striatum and mPFC of *Shank3* TG mice [14, 15]. Notably, however, in the striatum and mPFC of *Shank3* TG mice, ribosome-related genes were enriched in the down-regulated genes, but not in the up-regulated genes (Fig. 1h). Nevertheless, the ribosome-related core genes largely overlapped in all three brain regions (Fig. 1h, right panel and Additional file 2: Table 4), suggesting opposite directional changes of those genes in the hypothalamus compared with the striatum and mPFC of *Shank3* TG mice. Nevertheless, it should be considered that the ribosome-related genes in the *Shank3* TG hypothalamus were not DEGs. Therefore, we consider that the GSEA results may reflect some, possibly ribosome-related, functional changes in the *Shank3* TG hypothalamus which may lead to subtle, but overall, responses of ribosome-related genes. Our results show mRNA and protein expressions of *Shank3* in the hypothalamus, and the effect of mild *Shank3* overexpression on hypothalamic gene expression, thus providing a new platform to further investigate unique molecular and synaptic functions of Shank3 in the hypothalamus.

## Additional files

Additional file 1: Materials and methods including information about mice, RNA sequencing and analysis, RNA purification and qRT-PCR, and biochemistry and antibodies for Western blotting. (DOCX 39 kb)

Additional file 2: **Table S1.** Summary of RNA-Seq mapping results (Shank3 TG hypothalamus 10–12 wk). **Table S2.** List of DEG (Differentially Expressed Gene) from RNA-Seq analysis results (Shank3 TG hypothalamus 10–12 wk). **Table S3.** GSEA (Gene-Set Enrichment analysis) (Shank3 TG hypothalamus 10–12 wk). **Table S4.** Comparison of ribosome-related GSEA core enrichment genes (Shank3 TG Hypothalamus, mPFC and Striatum). (XLSX 101 kb)

## Abbreviations

Cav2: Caveolin 2
GO: Gene ontology
GPR85: G protein-coupled receptor 85
GSEA: Gene Set Enrichment Analysis
KEGG: Kyoto Encyclopedia of Genes and Genomes
mPFC: Medial prefrontal cortex
RNA-seq: RNA-sequencing
SHANK3: SH3 and multiple ankyrin repeat domains 3
TG: Transgenic
