# Supplementary material for: Transcriptome analysis of Shank3-overexpressing mice reveals unique molecular changes in the hypothalamus
Source: Mol Brain. 2018 Nov 27;11:71. doi: 10.1186/s13041-018-0413-4 (PMC6257967; doi:10.1186/s13041-018-0413-4)
Supplement: Supplementary file 1 — Materials and methods including information about mice, RNA sequencing and analysis, RNA purification and qRT-PCR, and biochemistry and antibodies for Western blotting. (DOCX 39 kb) [file 13041_2018_413_MOESM1_ESM.docx]

**Additional File 1**

**Transcriptome analysis of *Shank3*-overexpressing mice reveals unique molecular changes in the hypothalamus**

Chunmei Jin, Hyojin Kang, Shinhyun Kim, Yinhua Zhang, Yeunkum Lee, Yoonhee Kim and Kihoon Han

**Materials and Methods**

**Mice**

The *EGFP* (enhanced green fluorescent protein)*-Shank3* transgenic (TG) mice used in this study have been described previously [[1](#_ENREF_1), [2](#_ENREF_2)]. The male wild-type (WT) and TG mice were bred and maintained in a C57BL/6J background according to the Korea University College of Medicine Research Requirements, and all the experimental procedures were approved by the Committees on Animal Research at the Korea University College of Medicine (KOREA-2016-0096). The mice were fed and had access to water *ad libitum* and were housed under a 12-h light-dark cycle.

**RNA sequencing and analysis**

The mice (10 to 12-week-old male WT and *Shank3* TG) were deeply anesthetized with isoflurane and decapitated. The hypothalamus was dissected from each brain, immediately placed in RNAlater solution (Ambion), and stored at 4 °C overnight. The hypothalamus from 2 mice of same genotype were pooled to make one RNA sample, and a total three pairs of RNA samples (three WT and three *Shank3* TG, thus total six mice per each genotype) were processed for RNA sequencing. RNA extraction, library preparation, cluster generation, and sequencing were performed by Macrogen Inc. (Seoul, Korea). RNA samples for sequencing were prepared using a TruSeq Stranded mRNA LT Sample Prep Kit (Illumina) according to the manufacturer’s instructions. An Illumina’s HiSeq 2000 was used for sequencing to generate 101-bp paired-end reads (Additional file 2, Table 1). Raw data were submitted to the GEO (Gene Expression Omnibus) repository under accession number GSE120609.

Transcript abundance was estimated with Salmon (v0.9.1) [[3](#_ENREF_3)] in Quasi-mapping-based mode onto the Mus musculus genome (GRCm38) with GC bias correction (--gcBias). Quantified gene-level abundance data was imported to R (v.3.6.0) with the tximport [[4](#_ENREF_4)] package and differential gene expression analysis was carried out using R/Bioconductor DEseq2 (v1.19.11) [[5](#_ENREF_5)]. Normalized read counts were computed by dividing the raw read counts by size factors and fitted to a negative binomial distribution. The *P* values were first corrected by applying an empirical estimation of the null distribution using the R fdrtool (v.1.2.15) package and then adjusted for multiple testing with the Benjamini–Hochberg correction. Genes with an adjusted *P* value of less than 0.05 were considered as differentially expressed. Volcano plots were generated using the R ggplot2 (v.2.2.1) package.

The Gene Ontology (GO) and Kyoto Encyclopedia of Genes and Genomes (KEGG) pathway analyses were performed using DAVID software (version 6.8) [[6](#_ENREF_6)]. Mouse gene names were converted to human homologs using the Mouse Genome Informatics (MGI) database (<http://www.informatics.jax.org/homology.shtml>).

Gene Set Enrichment Analysis (GSEA) (http://software.broadinstitute.org/gsea) [[7](#_ENREF_7)] was used to determine whether a *priori*-defined gene sets would show statistically significant differences in expression between *Shank3* TG and WT mice. Enrichment analysis was performed using GSEAPreranked (gsea-3.0.jar) module on gene set collections H (Hallmark gene sets; 50 gene sets) and CP (KEGG; 186 gene sets) downloaded from Molecular Signature Database (MSigDB) v6.1 (http://software.broadinstitute.org/gsea/msigdb). GSEAPreranked was applied using the list of all genes expressed, ranked by the fold change and multiplied by the inverse of the *P* value with recommended default settings (1,000 permutations and a classic scoring scheme). The False Discovery Rate (FDR) was estimated to control the false positive finding of a given Normalized Enrichment Score (NES) by comparing the tails of the observed and null distributions derived from 1000 gene set permutations. The gene sets with an FDR of less than 0.05 were considered as significantly enriched.

**RNA purification and qRT-PCR**

Real-time quantitative reverse transcription PCR (qRT-PCR) was performed as described previously [[2](#_ENREF_2), [8](#_ENREF_8)]. Briefly, total RNA was extracted from the brain regions of WT and *Shank3* TG mice using an miRNeasy Mini Kit (Qiagen) according to the manufacturer’s instructions. Two micrograms of total RNA was used for cDNA synthesis using iScript™ cDNA Synthesis Kit (Bio-Rad). Target mRNAs were detected and quantified by a real-time PCR instrument (CFX96 Touch, Bio-Rad) using SYBR Green master mix (Bio-Rad). The results were analyzed using the comparative Ct method normalized against the housekeeping gene *Gapdh*. The primer sequences for real-time PCR are as follows:

Mouse *Shank3* forward 5’ TGGTTGGCAAGAGATCCAT 3’ (exon 6 of 22),

reverse 5’ TTGGCCCCATAGAACAAAAG 3’ (exon 7 of 22)

Mouse *Gpr85* forward 5’ ATGCAGCCGACAACATTTTGC 3’,

reverse 5’ CAGGTGGAGCCATTTTTGACA 3’

Mouse *Cav2* forward 5’ CTCAAGCTAGGCTTCGAGGA 3’,

reverse 5’ ACAGGATACCCGCAATGAAG 3’

Mouse *Gapdh* forward 5’ GGCATTGCTCTCAATGACAA 3’,

reverse 5’ CCCTGTTGCTGTAGCCGTAT 3’

**Biochemistry and antibodies for Western blotting**

Whole lysates of the mouse brain were prepared as described previously [[9](#_ENREF_9), [10](#_ENREF_10)]. Briefly, the hypothalamus of 12-week-old mice were homogenized in RIPA buffer (50 mM Tris-HCl pH 8.0, 150 mM NaCl, 0.1% SDS, 1% Triton X-100, 0.5% sodium deoxycholate) with freshly added protease and phosphatase inhibitors (Roche). Protein concentration was measured using Bradford Protein Assay (Bio-Rad). Brain lysates were heated in 1x NuPAGE LDS sample buffer (Invitrogen) containing a 1x NuPAGE reducing agent (Invitrogen). From each sample, 10~20 μg of proteins were loaded for Western blotting. The antibodies used for Western blotting were GAPDH (Cell Signaling, #2118), Homer1b/c (Santa Cruz, sc-20807), PSD-95 (NeuroMab, 75-028), Shank3 (Santa Cruz Biotechnology, sc-30193). Western blot images were acquired by ChemiDoc Touch Imaging System (Bio-Rad).

**References**

1. Han K, Holder JL, Jr., Schaaf CP, Lu H, Chen H, Kang H et al. SHANK3 overexpression causes manic-like behaviour with unique pharmacogenetic properties. Nature. 2013;503(7474):72-7. doi:10.1038/nature12630.

2. Lee B, Zhang Y, Kim Y, Kim S, Lee Y, Han K. Age-dependent decrease of GAD65/67 mRNAs but normal densities of GABAergic interneurons in the brain regions of Shank3-overexpressing manic mouse model. Neurosci Lett. 2017;649:48-54. doi:10.1016/j.neulet.2017.04.016.

3. Patro R, Duggal G, Love MI, Irizarry RA, Kingsford C. Salmon provides fast and bias-aware quantification of transcript expression. Nat Methods. 2017;14(4):417-9. doi:10.1038/nmeth.4197.

4. Soneson C, Love MI, Robinson MD. Differential analyses for RNA-seq: transcript-level estimates improve gene-level inferences. F1000Res. 2015;4:1521. doi:10.12688/f1000research.7563.2.

5. Love MI, Huber W, Anders S. Moderated estimation of fold change and dispersion for RNA-seq data with DESeq2. Genome Biol. 2014;15(12):550. doi:10.1186/s13059-014-0550-8.

6. Huang da W, Sherman BT, Lempicki RA. Systematic and integrative analysis of large gene lists using DAVID bioinformatics resources. Nat Protoc. 2009;4(1):44-57. doi:10.1038/nprot.2008.211.

7. Subramanian A, Tamayo P, Mootha VK, Mukherjee S, Ebert BL, Gillette MA et al. Gene set enrichment analysis: a knowledge-based approach for interpreting genome-wide expression profiles. Proc Natl Acad Sci U S A. 2005;102(43):15545-50. doi:10.1073/pnas.0506580102.

8. Kim Y, Zhang Y, Pang K, Kang H, Park H, Lee Y et al. Bipolar Disorder Associated microRNA, miR-1908-5p, Regulates the Expression of Genes Functioning in Neuronal Glutamatergic Synapses. Exp Neurobiol. 2016;25(6):296-306. doi:10.5607/en.2016.25.6.296.

9. Han K, Chen H, Gennarino VA, Richman R, Lu HC, Zoghbi HY. Fragile X-like behaviors and abnormal cortical dendritic spines in Cytoplasmic FMR1-interacting protein 2-mutant mice. Hum Mol Genet. 2015;24(7):1813-23. doi:10.1093/hmg/ddu595.

10. Han K, Kim MH, Seeburg D, Seo J, Verpelli C, Han S et al. Regulated RalBP1 binding to RalA and PSD-95 controls AMPA receptor endocytosis and LTD. PLoS Biol. 2009;7(9):e1000187. doi:10.1371/journal.pbio.1000187.
